# The Framing and Fashioning of Therapeutic Citizenship Among People Living With HIV Taking Antiretroviral Therapy in Uganda

**DOI:** 10.1177/1049732315597654

**Published:** 2015-08-05

**Authors:** Steve Russell, Stella Namukwaya, Flavia Zalwango, Janet Seeley

**Affiliations:** 1 University of East Anglia, Norwich, United Kingdom; 2 Uganda Virus Research Institute Research Unit on AIDS, Entebbe, Uganda; 3 London School of Hygiene and Tropical Medicine, London, United Kingdom

**Keywords:** HIV/AIDS, Africa, Sub-Saharan, illness and disease, experiences, coping and adaptation, health care, users’ experiences, self-care, adherence / compliance, research, qualitative

## Abstract

In this article, we examine how people living with HIV (PLWH) were able to reconceptualize or “reframe” their understanding of HIV and enhance their capacity to self-manage the condition. Two in-depth interviews were held with 38 PLWH (20 women, 18 men) selected from three government and nongovernment antiretroviral therapy (ART) delivery sites in Wakiso District, and the narratives analyzed. ART providers played an important role in shaping participants’ HIV self-management processes. Health workers helped PLWH realize that they could control their condition, provided useful concepts and language for emotional coping, and gave advice about practical self-management tasks, although this could not always be put into practice. ART providers in this setting were spaces for the development of a collective identity and a particular form of therapeutic citizenship that encouraged self-management, including adherence to ART. Positive framing institutions are important for many PLWH in resource-limited settings and the success of ART programs.

## Introduction

### Rationale and Objectives

HIV diagnosis can cause profound disruption to people’s lives and identity. The ability to come to terms with the condition, and shape a more positive identity, is shaped by the individual’s disposition, and the support they seek and receive from family, friends, and, depending on the context, a range of organizations. In this article, we analyze the role of HIV treatment providers in the lives of people living with HIV (PLWH) in Uganda who are on antiretroviral therapy (ART). We focus on how PLWH’s interactions with government providers, and The AIDS Support Organization (TASO), influenced their self-management of HIV. These organizations can shape how PLWH understand and cope with the condition, helping them to “reconceptualize” or “reframe” interpretations of the illness and how to self-manage HIV as a long-term condition (Abel, Rew, Gortner, & Delville, 2004; Watkins-Hayes, Pittman-Gay, & Beaman, 2012).

The influence of reframing processes is important for the success of ART programs because they rely on patients becoming active and effective self-managers. Many PLWH in Sub-Saharan Africa have been taking ART for several years, so on-going support from health providers to promote long-term self-management and adherence remains important (Beard, Feeley, & Rosen, 2009; Mbonye, Nakamanya, et al., 2013).

As access to ART expands in resource-limited settings, ART providers can benefit from better understanding of PLWH’s self-management of HIV, the constraints they face, and in particular the processes that help them to sustain engagement with the health system and their treatment. Trusting and productive relationships with health workers, which create this motivation to sustain self-management, are likely to be beneficial (Ware et al., 2009).

High levels of ART adherence in early HIV treatment projects in Africa have been explained by the concept of therapeutic citizenship (Nguyen, Ako, Niamba, Sylla, & Tiendrébégo, 2007). This concept refers to changes in people’s identity resulting from their biological condition and their social interactions with HIV organizations. These organizations exert power through the resources they offer and accepted discourses about how to tackle and live with HIV. Through these interactions, particular kinds of subjects are fashioned (Nguyen, 2005): People are encouraged to assert their rights, make claims for treatment, and are also expected to behave as “responsible” HIV citizens, including adherence to ART (Nguyen et al., 2007). The fashioning of therapeutic citizenship is a biosocial and biopolitical process: It involves power relations and techniques, including “moral frameworks,” to govern health behavior and manage patients (Cassidy, 2010; Mattes, 2011; Nguyen, 2005).

The fashioning of self-management and adherence is therefore the product of social interactions within support groups and health facilities, and social obligations to family and peers (Mfecane, 2011; Nguyen et al., 2007; Ware et al., 2009). Wider political contexts can also shape people’s sense of purpose to adhere (Kagee, Swartz, & Swartz, 2014; Kielmann & Cataldo, 2010).

In this article, the narratives of PLWH in Uganda on ART are analyzed to examine the influence of health care providers on their journey to becoming “responsible” self-managers of their condition. We examine their uptake of self-management messages, and consider whether there is a process of disciplining patients, or instead the creation of empowered patients with a positive discipline to self-manage. The analysis is informed by three broad concepts: chronic illness self-management (Schulman-Green et al., 2012); framing institutions and agents and their influence on people’s self-management (Watkins-Hayes et al., 2012); and therapeutic citizenship (Nguyen, 2005), which encapsulates how framing processes might fashion PLWH’s identity and responsibility for self-management and health, including drug adherence. Implications for ART programs are discussed.

Studies of people’s self-management of HIV on ART in resource-limited settings tend to focus narrowly on adherence to treatment. Only a few studies have looked at the broader range of social and emotional self-management and adjustment processes, which are important for sustained adherence (Martin, Kawuma, Zalwango, & Seeley, 2013; Mbonye, Seeley, Ssembajja, Birungi, & Jaffar, 2013; Roura, Wringe, et al., 2009; Russell & Seeley, 2010; Wouters, 2012). Very few examine how health worker messages and frameworks are absorbed by patients and affect their self-management (Allen et al., 2011).

### Self-Management, Framing Agents, and the Formation of Therapeutic Citizens

Self-management of long-term illness is a complex, dynamic, and interactive process, involving practical tasks, and psychological and social adjustments (Russell & Seeley, 2010; Schulman-Green et al., 2012; Sharpe & Curran, 2006; Swendeman, Ingram, & Rotheram-Borus, 2009). A systematic review of patient chronic illness self-management identifies three processes (Schulman-Green et al., 2012): the work of managing illness needs (recognizing symptoms, taking treatment, adopting healthier behaviors), activating resources (e.g., from the health system and social networks), and the more complex process of living with the condition. This latter group of self-management processes includes the emotional and cognitive work of adjusting to the condition and adjusting to a new sense of self (managing identity, dealing with stigma). These adjustment processes can be broadly defined as the incorporation of an illness and treatment into one’s life and identity (Russell & Seeley, 2010), and more specifically in psychological terms as “ . . .the process to maintain a positive view of the self and the world in the face of health a problem” (Sharpe & Curran, 2006, p. 1161).

Framing institutions, such as health care providers, and the framing agents working within them, can provide “ . . . language, adaptive skills and practical knowledge that shape how individuals interpret a new life condition . . . ” (Watkins-Hayes et al., 2012, p. 2030). Framing agents such as health workers are in a position of authority, which can have detrimental or productive effects for PLWH. They might “name and frame” HIV in ways that reinforce a negative conceptualization of oneself, by giving implicit messages about “needing to keep it secret” or “only having oneself to blame.” Health workers can also use their position of authority to control patients, for example, with threats of withdrawing treatment if patients are not obedient, passive, and compliant, fashioning a form of subordinated therapeutic citizenship.

Framing agents, however, can also enable people to reconceptualize their situation more positively, and cope and adjust more effectively. Several interrelated elements of the framing process can be distinguished:

- Provision of information about HIV and how it can be treated and managed, to help the patient reconstruct their perceptions of the condition, for example, that HIV is treatable and there is hope for the future;
- Supporting PLWH to develop conceptual frameworks and language, which help them adapt emotionally and cognitively, to make more sense of their situation, reduce negative emotions, and improve coping with HIV (Abel et al., 2004); and
- Advice about practical health-related self-management tasks, such as drug adherence, diet, or sexual behavior.

The concept of therapeutic citizenship also informs our analysis of people’s self-management of HIV. It is not a conventional understanding of citizenship, the relationship between individual and state (Nguyen et al., 2007), but rather a stateless citizenship with one’s identity fashioned around HIV organizational “assemblages.” The term “global assemblage” refers to the development organizations which have emerged around the HIV issue in recent decades and which produce interventions and discourses, and offer resources that fashion particular kinds of subjects (Nguyen, 2005). These citizens make claims on these assemblages, and seek and use treatment “as a set of rights and responsibilities” (Nguyen et al., 2007, p. S34). From the literature, several features of this concept are set out below:

- A near-death experience followed by HIV diagnosis and starting ART: biological and clinical processes that predisposed many to subsequent identity changes;
- Counseling processes and the fashioning of new perceptions of self and identities;
- A new sense of belonging to a group, developed, for example, at a support group;
- Strategies to access resources and treatment, to make claims on the wider global economy of the pharmaceutical market and HIV policy architecture;
- A process of articulating and fighting for rights; and
- A set of obligations and responsibilities of HIV citizenship, notably adhering to treatment and behaving as a “responsible” patient.

Therapeutic citizenship can therefore empower, by informing PLWH about their rights and exhorting them to claim them (Cataldo, 2008). It might also be fashioned, however, through the exertion of power in a Foucaultian sense, shaping patient identities to lead responsible and disciplined lives and be compliant with authorities, based on expectations about the “right” way to behave (Cassidy, 2010; Mfecane, 2011; Nguyen, 2005).

Different forms of therapeutic citizenship are fashioned in different contexts (Kagee et al., 2014). In settings where providers exert power to control patients, therapeutic citizenship might refer to the creation of docile patients at ART clinics (Mattes, 2011). Alternatively, the dramatic recovery of health on ART, a form of “resurrection” and second chance at life, might transform subjectivities and create more empowered HIV patients able to self-manage their condition in a disciplined way (Russell & Seeley, 2010).

A form of empowered therapeutic citizenship has been documented in South Africa (Fassin & Schneider, 2003; Kagee et al., 2014; Robins, 2006). Robins’s (2006) analysis in particular describes PLWH’s identity transformations and turn to HIV activism. This transformative process stemmed from their dramatic recovery on ART following “near biological death” and “social death” experiences, a “coming back from the dead,” which when combined with involvement in HIV organizations, had profound effects for the biological and social fashioning of PLWH into empowered, knowledgeable citizens and activists. They joined the Treatment Action Campaign and felt a strong responsibility to adhere to the hard fought-for treatment. Adherence to drugs was seen as a political statement and responsibility in this setting (Kagee et al., 2014).

## Method

### Research Design and Study Site

In 2011-2012, qualitative and quantitative data were collected for a study on PLWH’s coping and self-management processes on ART in Wakiso District, Central Uganda. The aim of the study was to develop conceptual and empirical understanding of PLWH’s self-management, and the factors that enable or hinder self-management processes. In this article, qualitative findings are presented on the role of health institutions and health workers in research participants’ self-management, from the participants’ perspectives.

Participants were recruited from three types of ART delivery site in the district: the HIV clinic at the government hospital in Entebbe; three government health centers that have referral links to Entebbe hospital; and the Entebbe branch of a well-established nongovernmental organization, TASO.

Ethical approval for the study was obtained from the Uganda Virus Research Institute (UVRI) Science and Ethics Committee and the University of East Anglia, United Kingdom. Overall approval was granted by the Uganda National Council for Science and Technology. Participants were recruited through patient registers at health care providers, so confidentiality was important to prevent the narratives getting back to providers, and to prevent the research from revealing a person’s HIV status in the communities where they lived. Confidentiality was achieved partly by anonymization of the data, using participant codes and pseudonyms at all times. The real names that matched the codes were kept on a list that was locked away securely at the Medical Research Council (MRC)/UVRI office in Entebbe. Interview transcripts were secured in locked cupboards and password-protected computers.

### Sample

To be eligible, participants must have been on ART for more than 1 year. A list of eligible patients was compiled from each facility. The lists were long, so to reduce the number from which we would purposefully select the sample, a systematic random sample was taken using intervals to generate twice the number of cases required. This provided selection choices and allowed for refusals or early dropouts. The lists were stratified by age and gender, and 42 participants were purposively sampled from the gender and age categories to ensure gender balance, a mix of ages, and a range of patient experiences and ART regimens. Four could not be interviewed successfully or more than once and were excluded from final analysis.

Many of the participants practiced cultivation as their main form of livelihood, but others were engaged in fishing, various forms of trade, as well as formal employment.

### Data Collection Measures

Participants were interviewed twice. The first interview was an unstructured life and illness history interview, which was conducted over two to three visits because of the wide-ranging and sensitive nature of some of the questions. These multiple visits allowed for iterative learning and the development of more focused follow-up questions. The first interview was not taped, because experience in this setting indicated that people are more open when not being recorded, especially in the first few interviews, but notes were taken and the narratives were written up in English by the interviewers.

The second interview, informed by the preceding life-history interview, was semistructured and taped, transcribed, and translated into English. It explored fully participants’ approaches to self-management since becoming HIV positive and starting ART. The use of several visits allowed a degree of rapport to develop, which led to rich discussions of participants’ experiences.

### Analysis

Qualitative data were organized using QSR Software Nvivo 9. To check analytical rigor, two researchers initially independently coded and checked results. Themes and subthemes for particular areas of analysis (e.g., stigmatization) were then discussed in more detail by the team members and these were agreed by the team after a 2-week analytical workshop. Themes were tested further by checking counterexamples and exceptions. Quotes used in the article are either the words of the participants or the interviewer’s words used in the write up of the first interview. Frequently repeated expressions across the interviews are not quoted but cited using single quotation marks.

## Results

### Participant Characteristics

Among the 38 participants (18 men, 20 women), 20 were aged between 26 and 40, 14 aged between 41 and 60, 2 were more than 60 years (1 man, 1 woman), and the 2 youngest participants were women (18–25 years). Thirteen were recruited from the government hospital, 11 from three referral health centers, and 14 from TASO Entebbe. More than half the participants had some primary education and the majority were married or in a relationship. Their main economic activities were subsistence farming, fishing, construction work, and petty trading.

### The Influence of Framing Agents on Illness Perceptions

The majority (31/38) had been experiencing illnesses, some very serious, before going for an HIV test. Some had lost their partner or a child to HIV. Their recollections of how they felt when they faced the health worker and received their test results revealed very negative illness perceptions. Common expressions included the following: “my life is over,” “you know you are going to die,” “who will care for the children,” “I will be rejected.” Some participants worried so intensely that they felt they would die of worry before HIV.

At TASO, first encounters with staff were usually with trained counselors and people were then assigned to a specific counselor responsible for their well-being. At government facilities, the participants first met a nurse, and services tended to focus more narrowly on providing ART orientation classes (held once per week for a month) and then the ART. Overall the information received by participants appeared to be similar across the two groups.

All 38 participants enthusiastically recalled how health workers changed their understanding of HIV. They learned how HIV attacks the immune system and that with treatment, the disease could be controlled and its consequences minimized. They were therefore helped to reframe HIV as a controllable not a terminal disease, with a future orientation extended from months to years, but that this depended on adherence to the treatment and advice about staying healthy.

New knowledge and illness perceptions enabled the participants to reappraise their situation and begin a process of moving from a state of despair to a more positive outlook for the future, a process summarized well by a female participant: “My heart became strong because of the things that we were told by the health workers during counseling” (woman, age 27). Counseling gave the participants hope:

. . . I do not spend time thinking about how I am sick, no, no, no! That I am going to die. That was in the beginning when I used to get many thoughts. . . . Now I have moved away from that stage and counseling helped me to overcome that. (Man, age 44).

For several participants, initial side effects on ART were serious and caused doubts about what they had been told. The health workers’ role at this time, to encourage and assure them this was normal, was particularly important:

When I called my counselor and told him how the drugs were affecting me . . . many things were happening . . . like bad dreams, my private parts had swollen . . . and I felt like life was coming to an end. My counselor told me to go to TASO . . . . When I reached the counselor he encouraged me . . . he told me to drink a lot, eat well until the body gets used to the drug. He told me that the beginning is always hard but I will adjust. (Man, age 38).

Participants were receptive to health worker advice because it was encouraging and met their great need for a positive message. Two other recurring experiential themes in the narratives explain PLWH’s positive reappraisal of their situation. The first was their recovery on ART: They could feel for themselves how HIV was treatable and controllable. The second experience, frequently and powerfully expressed in the narratives, was the importance of “seeing and being with others who had HIV and looked well,” identifying with these others’ positive experiences. Seeing others was also motivational because it helped them see they were not alone.

### New Concepts and Language for Coping

Participants talked a lot about the ways health workers had helped them cope psychologically. One of the first things participants were told was “not to worry, because this will make you sick.” This was a simple message and a hard one to put into practice, but participants appeared to have strived to work at it:

They taught me that if I worried too much I would lose weight and get problems so I cast my worries aside. . . .We were told that when you are sick that should not stop you from being happy so I try to be happy all the time. (Woman, age 27).

Health workers provided ways of thinking about HIV, which supported a more positive identity and psychological coping, and helped participants move toward accepting their condition. Counseling helped participants develop concepts and a language that “normalized” HIV. It was reconceptualized as a normal disease, “like many other diseases,” and also just one of many causes of death: “you can die from many other diseases and many other things”; “death comes to us all, so how is HIV any different?”

HIV was also reframed as a normal disease through reference to its wide prevalence and effects across the community: “It is difficult to find a family that has not been affected by HIV in this generation” (woman, age 43). Health workers told them from the beginning that “you are not alone, look around you,” and all the participants drew on this language, using the phrases “I am not the only one” and “I share this problem with many others.” This reconceptualizing of HIV as a normal disease helped participants reappraise their identity as a “normal” person.

For stigmatizing illnesses such as HIV, framing agents have the potential to offer an alternative language for thought and speech to assist coping and resist stigmatizing discourses, especially for marginalized groups (Watkins-Hayes et al., 2012). Health workers encouraged resistance thinking by making comparisons with others in the community. The majority of participants used a “Them and Us” language of comparison between themselves and “the many others who had not gone for a test and were ignorant of their status.” They viewed themselves, individually and as a group of patients, as knowledgeable and responsible citizens who had taken action to get tested, gain control of the situation, and not harm others:

They (the health workers) told us that we were better than those who had not bothered to know their status, that we were better than those that were laughing at us . . . saying that the (TASO) motorcycle has come to your home . . . they are also sick but do not take the responsibility to get tested so they don’t know their status. That is what made me brave. (Woman, age 27)

This categorization of themselves as “responsible” citizens compared with “others” who are irresponsible had become an ingroup identity, which enhanced their sense of moral upstanding and self-esteem. Such labeling into “good” and “bad” can be divisive and be the basis of stigmatization, for example, the division of HIV patients into “good” (adherers) and “bad” (nonadherers) can be applied as part of a therapeutic citizenship discourse to sanction nonadherence. This particular “Them and Us” distinction, however, was a defense of all HIV patients and a form of resistance thinking against a dominant group in the community who seek to stigmatize: They were not seeking to divide PLWH into good and bad patients. The overwhelming sentiment in the narratives was an empathy and solidarity felt for those who struggled to adhere.

### The Fashioning of “Responsible HIV Positive Citizens”

#### Health worker instructions: A framework for managing HIV

Participants described the advice and instructions given by health workers to sustain health. Prominent messages absorbed by participants were as follows: first and foremost, to adhere to medication and set times each to take the pills; second, to eat nutritious food, boil drinking water, and stop alcohol consumption; and third, “responsible” sexual behavior. Regarding sexual behavior, messages targeted particularly at men were as follows: “do not be promiscuous,” “be faithful to your partner,” and “give up on multiple partners.” Messages targeted toward women were “abstain (if you can),” “reduce sexual activity,” “avoid pregnancies,” and “giving birth weakens health.”

The same package of advice and instructions was described by those using TASO and government providers, and is similar to guidelines reported elsewhere in Uganda (Allen et al., 2011). Some reflect moral discourses about the negative effects of sex for PLWH (abstain from sex, do not have children). Not all health worker messages, therefore, were promoting a normalization of HIV in the era of ART, and could have potentially caused continuing self-stigmatization.

Participants described the package of instructions as a useful framework for self-management and living with HIV, which had enhanced their well-being:

I knew that I was finished and was just waiting for the day I would die. But sticking to the words of the *basawo* (health workers) helped me . . . and that is why I look fine now. (Man, age 45)

Health workers were viewed to have authoritative knowledge about HIV and participants had been willing to accept the instructions: They were not negotiated with health workers to arrive jointly at a treatment plan. Health workers’ authority, however, did not appear to have fashioned disciplined “docile bodies or undermined participants” sense of agency. On the contrary, they argued the framework had been a tool to support their own adjustment to life on ART; it gave them back a sense of control over their lives and had been a key factor in their recovery. The instructions, delivered in a respectful way in a supportive environment, had assisted their own self-management processes, summed up nicely by this female patient: “I help myself the most with my illness, because I follow the instructions of the health workers” (woman, age 29). A male participant articulated well the sense of control and security he felt from the instructions:

I am in control of the illness when I respect what the health workers tell me to do . . . That is why you see me build and going forward and when I look back, before I started on ARVs, I used to be disturbed by diseases . . . I said that I am going to die. But now, I do not have that thought. If you ask me my dreams now or what I am planning, I can tell you that I am going to plant a mango tree and I will be able to eat fruits from it. (Man, age 38)

Across all the interviews, on only two occasions was an health workers’ abuse of their authority reported, which was the indirect threat to withdraw treatment as a means of social control over a “difficult” patient. For example, a male participant described a fellow patient who had talked about his rights at the clinic. The health worker had told the patient “if you know more than us, go somewhere else, it will be easier for you.” None of the participants themselves had experienced this threat. One male participant said he had once missed an appointment, and was chastised for this, but that he understood why keeping appointments was important, and on that occasion, he received further counseling and was given the medication.

All the advice discussed by participants in their narratives appeared to have contributed to the fashioning of their responsible citizenship as a person living with HIV; the two most strongly and frequently discussed aspects of this citizenship are examined in more detail below.

#### The responsibilities of adherence and promoting health

Adherence messages had been understood by all the participants, and all except one stated they were adhering to treatment. An acceptance of the medicalization of their lives was evident: taking the drugs was ingrained into their minds: “I take my drugs without a reminder, I am programmed to take them” (woman, age 26).

High levels of adherence were indicated by the health of the participants, of which they were proud, saying it was one of their greatest achievements. They used metaphors such as “drugs are my food” and “I respect the drugs, they are like my mother and father.” However, one man was not adhering to treatment, possibly because of mental illness and lack of money for the food he said he needed to take with the drugs. Another man said he did not take an evening tablet 4 times each month, because on those nights he went out drinking with friends and was worried about mixing the drugs with alcohol. Only in serious situations (relating to bereavement and migration) were two female participants forced to stop taking the drugs for a period.

Messages about eating a good diet, drinking clean water, and hygiene had also been absorbed into the language of self-management. These were relatively easy messages to accept but sometimes hard to act on because of budget constraints. Income poverty meant many participants could only eat one main meal a day, and 4 of 20 women and 5 of 18 men struggled for enough food on a daily basis.

A frequent expression used, “I take the drugs even though I cannot eat the recommended food,” indicates participants were determined and committed to adherence: “Even though I do not have something to eat because of the scarcity of money, I swallow the drugs because I know my life depends on them” (man, age 32).

Men and women were told to stop drinking alcohol and smoking. Men’s narratives were more preoccupied with the issue of stopping drinking than women’s narratives, although women might have felt inhibited to talk about alcohol consumption because drinking is a socially undesirable behavior for women. The majority of men said they had stopped drinking because it caused them to forget the drugs, and health workers had told them that alcohol hinders the efficacy of ART.

Drinking alcohol was integral to men’s social lives: sharing a drink with friends was an important part of their self-management, of sustaining friendships, and living with HIV. Although all the men (and women) acknowledged the need to stop drinking, and some men had completely stopped, several men’s interviews indicated that “stopping” actually meant a substantial reduction in the frequency of drinking and binge drinking:

Every Wednesday and Sunday, I go to thank God for helping me stop drinking alcohol. I realized that alcohol would make me forget to take the drugs and it also contains acids which might not go well with the drugs. I used to drink all types of alcohol . . . I sometimes feel like taking *malwa* (local brew made of millet), and if I get *omulamba* (made from sorghum) I drink it because it is light and very delicious. (Man, age 43)

Overall, participants’ self-management processes were indicative of a desire to change behavior and follow the instructions. Often the language of trying to change rather than achieving it was used: “I eat a good diet; I try to avoid drinking alcohol” (woman, age 29).

#### Responsible sexual behavior

Participants understood that changes to sexual behavior were important, if not central, aspects of their self-management, a serious responsibility to protect their own health and the health of others. However, the constraints around sexual behavior change faced by PLWH, and aspirations for children, which they might need to negotiate in a partnership, are well documented in Uganda (Allen et al., 2011; Homsy et al., 2009; Martin et al., 2013; Mbonye, Nakamanya, et al., 2013; Seeley et al., 2009).

Sexual behavior messages had an obvious biomedical logic, but similar to the instructions about drinking, the messages from health workers also contained a moral logic, because being sexually “responsible” and thinking of others’ health (e.g., abstaining, not having multiple partners) is what one should morally do in this setting. Sexual behavior change messages were also gendered: Their content appeared to be targeted differently toward men and women, and generally required different types and degrees of behavior change for men and women. Messages targeted behaviors more usually identified with men: promiscuity and multiple partners.

Sexual behavior messages had been understood and absorbed, and the narratives indicated that participants did their best, in their circumstances, to follow advice. Decisions about sex, however, were constrained by notions of masculinity and womanhood, norms and desires to have children, and economic necessities. Half the women said they were now following advice to abstain after losing their husband to HIV, a decision made easier because they had already had children. Some women also emphasized the positive decision to “give up on men,” to avoid the trouble that men cause, as well as to protect their physical health. Some explanations for abstinence also exemplified the moral rather than biomedical logic of health worker advice; for example, several women’s narratives were similar to those of this woman: “I stayed away from men because we were told by counselors that if you have frequent sex you die quickly” (woman, age 43).

Women in partnerships were negotiating the rules to fit their constrained circumstances. Four were with their partner from the time of diagnosis, and six had found new partners since diagnosis (two had not disclosed their status to their partner). Their self-management decisions relating to sex were constrained because of economic dependency on their partner and the strong expectation that they should have children. For example, these women were either using condoms inconsistently or had stopped because it was difficult to sustain condom use in a long-term relationship. Two of the women had had a child since starting on ART and one was pregnant at the time of research.

Men were also aware of the behavior changes expected of them. Over the course of three or four meetings with the men, it was evident that they had put some of these changes into practice, to differing degrees. Notably none talked about abstinence, but most said they were now faithful to their one partner and used condoms with that partner:

I have sex with my wife but not on a regular basis. We are told that frequent sex can make you weak. They also told us to use condoms during sex to prevent new infections and unwanted pregnancies. (Man, age 45)

Men’s responses overall can be summarized as “abiding by most of the rules, most of the time,” and choosing to interpret them in ways that enabled them to sustain pleasure and existing relationships. For example, one man reported that he had stopped “partying and seeking women,” but continued to be with his two wives, using condoms with both partners. Some men did continue to have sexual partners outside marriage, although they “no longer went with so many women.” For example, a man who had had children with three women and now lived with only one wife, did not use condoms with his wife, but said he did so when he had sex with other women. Some men also reported that their partner was putting pressure on them to have a child, which affected safe sex practices.

### The Collective Fashioning of Therapeutic Citizens

The narratives of this group of PLWH indicated that their motivation and sense of responsibility for HIV self-management had been socially fashioned within the HIV clinic through their relationships with health workers and fellow patients.

What has helped me most is the counseling that I have been getting from the *basawo* (health workers). . . . They give us encouraging words that make us strong . . . it brings hope to the heart. (Man, age 39)

He came to realize that the doctor at the health center was the person who would help him live longer . . . he is the one who took him through counseling. . . . The doctor is a very caring person. When he meets him he asks him about everything and also advises him on how to deal with challenges. (Man, age 43)

Participants described how well they were cared for, treated, and respected at TASO and at the government hospital or health centers (with one or two exceptions), for example: “When I went to TASO, I felt like I was with my friends” (woman, age 26). The care and support at TASO was a particularly powerful narrative, and illustrated the profound influence of TASO on people’s lives after HIV. Good interpersonal quality of care at the government hospital HIV clinic was also frequently reported, which helped to sustain patient engagement with the health system: “The health workers at the hospital clinic were very warm and welcoming, which gave her courage to remain there and keep going back” (woman, age 58).

A second social process contributing to the participants’ responsible self-management was the fashioning of a sense of group membership, a collective identity, and shared responsibility for the “fight” against the challenges of HIV. The participants spoke about TASO and government facilities as a space, and their regular appointments as a dedicated time, in which they could have caring interactions not just with staff but also with other PLWH, to share experiences and encourage each other: “We sit and converse as a family” (woman, age 58). The clinic was a space where new friends, support networks, and identities were forged, where participants got encouragement and found motivation to start or continue to manage their illness: “The thing that has helped me is the fact that people comfort me when I go to the clinic and they give me great advice” (woman, age 27). Female participants talked more about the support found through their new friendship groups at service providers:

This is a great feature about meeting people at the clinic . . . we also give each other a call to check up on each other and things like that . . . we are encouraged because we are not alone; so many others are ill. (Woman, age 33)

Sharing experiences helped create a sense of membership and relatedness, of belonging and solidarity:

When we are gathered at the clinic, we benefit a lot. This situation unites us and we are the same. In fact, we call ourselves members; so when we meet, we simply greet each other with “hello member.” It is as if we are in a club. (Man, age 44)

There are people that we meet at the clinic . . . they live around here, I meet them and we greet one another. They might ask you for money and you help them knowing that you are a family. (Man, age 45)

Among the participants this sense of “membership” in a therapeutic community strengthened the motivation to work at self-management, and a sense of obligation or responsibility to act as a role model for and to support others in the community. Health workers had helped to build this identity, asking established patients to offer peer support to new arrivals at the clinic:

(A) woman was at the clinic and she cried so much. . . . I asked her what the matter was and she told me to leave her alone. . . . I told her to tell me what the problem was so that I could help her. She told me that she did not know that her husband was infected, that he simply brought her for testing and she was told that she has HIV. I then told her that since she was here (TASO) she was going to be in good health. . . . I told her that once you get to TASO and they give you drugs, you follow the instructions, stop worrying then all the things that were making you cry will go away. . . . After thinking through what I had told her she thanked me and stopped crying. We then started talking and watching TV and I told her that all those people that you see are sick even though they are looking good. I told her that she was lucky to know her status and start on treatment immediately. You will be healthy, able to have children, educate them and live for another twenty years. . . . The woman became so happy. (Woman, age 27)

## Discussion

### The Importance of Framing Institutions for PLWH in Uganda

Government and NGO HIV treatment providers gave the PLWH in our study information, concepts, language, and skills that enabled them to reframe their situation more positively and build self-management pathways. Health worker influence extended into a wide range of practical, social, and psychological self-management processes identified in other studies (Schulman-Green et al., 2012; Swendeman et al., 2009).

The sample for this study is unlikely to be typical of all PLWH in this setting. This group had tested, started treatment, and the majority were adhering to ART. They were open enough about their status to be willing to participate in the research. Another limitation was the inability to observe all self-management behaviors. However, the main purpose of this article is not to measure behavior objectively, but to examine whether participants had integrated framing messages into their concepts and language about self-management, and explore whether their self-management was linked to their sense of belonging to a community of therapeutic citizens. Our method of multiple interviews, the building of trust, and observations of the home environment give us confidence that the narratives did reflect participants’ self-management identities and behaviors.

Positive patient–provider relationships can improve HIV self-management and health outcomes (Johnson et al., 2006). Our findings show that good health worker–patient relationships did build trust and patient receptiveness to health worker framing messages. Information and positive encouragement enabled participants to reconceptualize their situation with optimism and played an important role in early acceptance processes. Framing processes also offered useful concepts and language for emotional and cognitive coping, including disease normalization and resistance thinking that helped in the reduction of self-stigmatization. Similar processes of HIV normalization and reductions in self-stigma following ART and counseling have been found in the region (Roura, Urassa, et al., 2009).

Advice about practical self-management tasks was absorbed although could not always be put into practice. Notably, 37 of 38 participants had become proud self-managers of HIV on ART. The frameworks provided by TASO and government services provided a helpful structure to regain control over the disease and their life. Participants had made their own positive decisions to follow the rules, which made them feel good physically and mentally, it gave them a sense of achievement and contributed to a heightened sense of well-being, which we have reported elsewhere (Martin, Russell, & Seeley, 2014).

The participants also felt being able to make their own decisions about when and how to transgress the behavioral boundaries for self-management set by the health workers. They were adhering to the drugs, but over time, they negotiated the rules to suit their needs, especially around decisions to drink socially, about condom use, and whether to try and have children. Similar patient negotiations with the rules of “being a good therapeutic citizen,” and occasional transgressions of these rules despite sticking to the broad remit, have been identified elsewhere in Sub-Saharan Africa (Allen et al., 2011; Mbonye, Nakamanya, et al., 2013; Mfecane, 2011).

In our study setting, this sense of agency was enabled by trusting relationships built with health workers, and in general (but not always) the good interpersonal quality of care experienced at providers, especially at TASO. They did not fear going to the clinic, nor did they fear sanctions being used against them if they missed an appointment or treatment.

The health worker–patient relationship was therefore productive for this particular group of PLWH, even though it was hierarchical. Participants valued instructions, as long as they came within a caring context of respectful relationships. They provided order and a sense of direction, and counterintuitively allowed them to develop a sense of control over their health and lives.

Our findings contrast with another study in the region, set in urban Tanzania, which concluded that a national government ART program was, in one particular ART delivery setting, creating a different form of therapeutic citizenship, characterized by disempowered patients who were controlled and disciplined by health providers (Mattes, 2011). The contrasting conclusions from this study might stem from several differences between the study settings. First, in the Tanzanian study health workers at the HIV clinic dealt with patients in an authoritarian and controlling manner, and threatened to remove patients from the treatment program if they did not follow the rules, “to control and discipline the conduct of rapidly growing patient masses” (Mattes, 2011, p. 160). This different patient–provider environment and relationship was partly the product of the pressures and stress to keep up with increasing patient numbers in a large overcrowded hospital. Second, in the Tanzanian study, “patients belonged to the lowest societal strata” (Mattes, 2011, p. 168), and the article suggests that they were not educated or “scientifically informed.” In our study, the participants were from more mixed social, educational, and income strata, and were convinced by the logic of biomedicine. They exerted agency in their self-management and had decided to be disciplined with their adherence, while negotiating the health promotion rules when needed.

### The Formation of Therapeutic Citizens

Participants’ personal engagement with self-management and ART adherence was socially fashioned. The narratives showed that health care providers were spaces for the development of a form of therapeutic citizenship, founded on common illness experiences and social interactions with health care workers and fellow patients. These experiences contributed to a sense of group membership, shared identity and responsibility, and set of practices for our study participants. This perspective “ . . . contrasts with approaches that examine adherence as the behavioral outcome of individual-level determinants” (Nguyen et al., 2007, p. S34).

The main characteristics of this form of therapeutic citizenship were as follows:

- Motivation to learn and acquire new knowledge;
- Acquisition of new concepts and languages about HIV, and resistance thinking;
- Acceptance of a medicalization of life and adherence to treatment
- Taking responsibility for one’s condition;
- Responsibly self-managing one’s health;
- Having a sense of responsibility for the health of others;
- Raising awareness and supporting other PLWH; and
- Having a collective sense of belonging to a wider community of PLWH.

The concept of therapeutic citizenship is nuanced, and people’s self-fashioning of new identities must be contextualized (Kagee et al., 2014; Mfecane, 2011). In this Ugandan study setting, where traditions of political activism against the state are scarce, and now that ART is being delivered to more than half the population needing it through conventional clinic environments, therapeutic citizenship does not refer to a radical process of empowerment and political activism, as identified in South Africa (Robins, 2006), nor does it refer to the degree of social control reported in Tanzania (Mattes, 2011). Rather, in our Ugandan study site, therapeutic citizenship refers to an emergent HIV community characterized by a sense of self-efficacy, commitment to self-management, and a desire to take ownership of one’s health.

The remarkable similarity of the narratives, about their journey from illness to recovery, and what had changed in their lives, to some extent, reflects the narrative as a device that people use to transform illness from an individual to a collective experience (Hydén, 1997). It reflects the shared experiences of the participants, shaped in the same social context and similar institutional environment: They underwent common framing processes and acquired the same concepts and language about recovery and self-management.

The harsh economic and social circumstances faced by many participants in this setting mediated their self-management strategies and the fashioning of their therapeutic citizenship. Income poverty, for example, meant people could not eat the food they had been advised to eat. What was so revealing from the narratives however was that in the face of these difficulties or constraints, the PLWH remained determined to work to make a success of their self-management and adhere to the drugs. For them, the main challenge was not HIV, but the hard struggle of poverty.

As Mattes’s (2011) study demonstrated, and as Moyer and Igonya (2014) have recently reminded us, although treatment is becoming more readily available, access to “good care,” meaning the nonbiomedical support many marginalized PLWH continue to need, is not. Given the burden of HIV in Sub-Saharan Africa, and the need for lifelong motivation to sustain ART adherence, there remains a need to expand low-cost interventions that encourage PLWH to talk, learn, and reconceptualize the disease, to help their psychological adjustment, reduce stigmatization, and promote their sense of control to self-manage their condition. Our findings demonstrate the need to sustain care-oriented community-based organizations, such as TASO, which foster caring and enabling relationships with PLWH.
